# Cognitive and Neural Mechanisms of Social Communication Dysfunction in Primary Progressive Aphasia

**DOI:** 10.3390/brainsci11121600

**Published:** 2021-12-01

**Authors:** Zoë-Lee Goldberg, Hashim El-Omar, David Foxe, Cristian E. Leyton, Rebekah M. Ahmed, Olivier Piguet, Muireann Irish

**Affiliations:** 1 Brain and Mind Centre, The University of Sydney, Sydney, NSW 2050, Australia; zoe.goldberg@sydney.edu.au (Z.-L.G.); hashim.el-omar@sydney.edu.au (H.E.-O.); david.foxe@sydney.edu.au (D.F.); cristian.leyton@sydney.edu.au (C.E.L.); rebekah.ahmed@sydney.edu.au (R.M.A.); olivier.piguet@sydney.edu.au (O.P.); 2 School of Psychology, The University of Sydney, Sydney, NSW 2006, Australia; 3 School of Medical Sciences, University of Sydney, Sydney, NSW 2006, Australia; 4 Memory and Cognition Clinic, Department of Clinical Neurosciences, Royal Prince Alfred Hospital, Sydney, NSW 2050, Australia

**Keywords:** social cognition, language, frontotemporal dementia, Alzheimer’s disease, thalamus, frontal lobe

## Abstract

Mounting evidence suggests that, in parallel with well-defined changes in language, primary progressive aphasia (PPA) syndromes display co-occurring social cognitive impairments. Here, we explored multidimensional profiles of carer-rated social communication using the La Trobe Communication Questionnaire (LCQ) in 11 semantic dementia (SD), 12 logopenic progressive aphasia (LPA) and 9 progressive non-fluent aphasia (PNFA) cases and contrasted their performance with 19 Alzheimer’s disease (AD) cases, 26 behavioural variant frontotemporal dementia (bvFTD) cases and 31 healthy older controls. Relative to the controls, the majority of patient groups displayed significant overall social communication difficulties, with common and unique profiles of impairment evident on the LCQ subscales. Correlation analyses revealed a differential impact of social communication disturbances on functional outcomes in patient and carer well-being, most pronounced for SD and bvFTD. Finally, voxel-based morphometry analyses based on a structural brain MRI pointed to the degradation of a distributed brain network in mediating social communication dysfunction in dementia. Our findings suggest that social communication difficulties are an important feature of PPA, with significant implications for patient function and carer well-being. The origins of these changes are likely to be multifactorial, reflecting the breakdown of fronto-thalamic brain circuits specialised in the integration of complex information.

## 1. Introduction

Primary progressive aphasia (PPA) refers to a collection of diverse neurodegenerative clinical syndromes characterised by progressive deterioration of language and speech functions. Current diagnostic criteria recognise three PPA variants based on distinct profiles of linguistic changes, distribution of brain atrophy and underlying neuropathology [1]. These variants are semantic variant PPA (referred to here as semantic dementia (SD)), non-fluent/agrammatic variant PPA (or progressive non-fluent aphasia (PNFA)) and logopenic variant PPA (or logopenic progressive aphasia (LPA)), each of which is characterised by differential patterns of language and motor speech dysfunction. SD is defined by the progressive loss of conceptual knowledge, which impairs both receptive and produced language in the context of relatively intact fluency, phonology, and syntax. Impoverished language is also a prototypical feature of LPA, manifesting in slowing of spontaneous speech, phonological errors and paraphasias, sentence repetition and word-finding difficulties. Finally, PNFA is typified by the progressive disruption of language fluency, resulting in hesitant, distorted speech and diminished grammatical complexity.

Over the past decade, significant progress has been made in understanding the variable profiles of language and motor speech dysfunction exhibited by PPA syndromes as well as their potential neuroanatomical and pathological signatures (reviewed by [2,3]). Despite such advances, it remains challenging to differentiate between PPA variants in the clinical setting, resulting in delayed diagnosis and poor patient prognosis [4,5,6]. This challenge, in part, reflects the fact that in parallel with well-defined changes in language, PPA syndromes frequently present with co-occurring non-linguistic cognitive deficits [7,8]. While these non-linguistic cognitive deficits are generally proposed to scale with disease severity, mounting evidence suggests highly variable and overlapping cognitive profiles, independent of primary language dysfunction [9]. One domain that has received increasing attention in this regard is that of social cognition [10].

Changes in social and interpersonal function are increasingly recognised as early features of PPA that can be intertwined with or independent from the canonical linguistic and motor speech changes in these syndromes. Mounting evidence points to marked socioemotional disturbances across the majority of PPA variants, with the suggestion that many of these symptoms emerge in parallel with, and not secondary to, their language and speech production difficulties (reviewed by [10]). A growing literature indicates striking impairments in emotion processing in PPA [11,12,13,14], although mixed findings have been reported in relation to LPA [15]. Similarly, theory-of-mind impairments are now understood to emerge as a standalone feature in SD [16,17] and PNFA [18], while diminished capacity for empathy is also widely observed in PPA syndromes ([13,19,20], but see [21]). This co-occurrence of social cognitive and language disturbances in PPA has important implications in terms of functional outcomes for patients, as it can further affect the ability to communicate meaningfully, and may disrupt the capacity to form and sustain interpersonal relationships [22].

In this context, pragmatics represent an important aspect of communication to explore. Pragmatics refers to the use of natural language, in terms of literal and non-literal aspects of communicated meaning, within particular interactional contexts [23,24]. To bridge the gap between what is said and what is actually meant, pragmatics are posited to require some form of inferential process and thus are viewed as essential to social communication [25]. Social communication refers to the use of language in social contexts and relies on attuning one’s linguistic behaviour and intent to the specific demands of a given context, inferring the speaker’s intended meaning to provide the appropriate information and engaging in an array of coordination processes, including turn-taking, topic maintenance and terminating at the appropriate juncture [26]. The multifaceted nature of social communication has been suggested to draw upon a distributed brain network, comprising distinct dorsomedial prefrontal, anterior temporal and inferior parietal cortices, and their respective connections, to coordinate the representation of informative actions, communicative intentions, processing of lexical/semantic information, syntactic analysis and pragmatic integration [27]. Importantly, many of these regions harbour brain atrophy from early in the PPA disease course, suggesting that social communication would be deleteriously affected in these disorders. Despite some evidence of decline in pragmatics in healthy aging [28] and mild cognitive impairment (MCI; [29]), it remains unclear how social communication is affected in PPA or neurodegenerative disorders more broadly.

The objectives of the current study were threefold. First, we sought to characterise the nature of social communication dysfunction across PPA syndromes (SD, LPA, PNFA) using a validated tool, the La Trobe Communication Questionnaire (LCQ; [30]), and to contrast profiles of impairment in PPA with that of Alzheimer’s disease (AD) and the behavioural variant of frontotemporal dementia (bvFTD), two dementia disorders in which language and socioemotional functions are differentially impacted [31,32,33]. Second, given that pragmatic disruption in clinical disorders is associated with negative effects on social relationships and quality of life [34], we sought to establish the impact of social communication dysfunction on patient functional outcomes and carer well-being. Finally, to provide a comprehensive understanding of the neurobiology of social communication, we conducted voxel-based morphometry analyses on structural brain scans to determine the neural signature of social communication dysfunction using a transdiagnostic approach.

Given the profound nature of language and motor speech difficulties in PPA, we hypothesised that social communication disturbances as measured by the overall score on the LCQ would be present across the majority of PPA syndromes. Importantly, we predicted that distinct profiles of social communication would be evident across the LCQ subscales and that the severity of social communication dysfunction would scale with the overall level of functional impairment in patients and the degree of burden reported by carers. Finally, in terms of underlying neural substrates, we predicted that the severity of social communication dysfunction would correlate with the magnitude of atrophy in frontoparietal brain regions implicated in speech, language, and social inference [27].

## 2. Materials and Methods

### 2.1. Participants

A total of 108 participants took part in this study, of which 12 patients with a clinical diagnosis of logopenic progressive aphasia (LPA), 11 patients diagnosed with semantic dementia (SD; 4 cases with right-sided SD) and 9 patients diagnosed with progressive non-fluent aphasia (PNFA) [1] were contrasted with 19 clinically probable Alzheimer’s disease (AD) [35] and 26 clinically probable behavioural-variant frontotemporal dementia (bvFTD) [36] cases. A comparison group of 31 healthy older controls was recruited. All participants were seen through the FRONTIER frontotemporal dementia research group in Sydney, Australia. Diagnoses were established by multidisciplinary consensus between a senior neurologist (RMA), a clinical neuropsychologist and an occupational therapist in line with current consensus criteria. All participants underwent comprehensive clinical investigation and neuropsychological assessment along with structural neuroimaging. Disease duration was estimated using years elapsed since reported onset of symptoms. Disease staging was determined using the Frontotemporal Dementia Functional Rating Scale (FRS) [37], a dementia staging tool sensitive to changes in functional abilities, activities of daily living and behavioural symptoms. Carers rated their own perceived levels of burden using the Zarit Burden Interview (ZBI) [38].

Exclusion criteria for all participants included prior history of stroke, epilepsy, alcohol and other drug abuse, significant traumatic brain injury, other primary neurological, psychiatric or mood disorders and limited English proficiency.

Ethics approval for this study was granted by the University of New South Wales and the South Eastern Sydney Local Health District human ethics committee. All participants, or the persons responsible for them, provided written informed consent in accordance with the Declaration of Helsinki. Participants were provided with information sheets and consent forms in advance of their research appointment to provide time to review the study aims and seek clarification on any aspects of the proposed research. On the day of testing, the information sheets and consent forms were reviewed again in person with a member of the research team to allow further opportunity for questions and discussion.

### 2.2. Cognitive Screening

Global cognitive functioning was determined using Addenbrooke’s Cognitive Examination, Third Edition (ACE-III; [39,40]), a test of cognitive function covering attention, memory, verbal fluency, language, and visuospatial abilities. In addition, participants completed a comprehensive battery of neuropsychological tests assessing integrity of the main cognitive domains. Attention was assessed using Digit Span forwards [41]. Executive function was measured using the Trail Making Test (Part B-A) [42], while non-verbal episodic memory was assessed using the Rey Complex Figure [43], from which we calculated a percentage retained score (3 min recall/Copy × 100) (see also [44]). Language ability was assessed using the naming, comprehension, semantic association and single-word repetition subtests of the Sydney Language Battery (SYDBAT; [45]). Finally, inhibitory control was measured using the Hayling Sentence Completion Test [46].

### 2.3. Assessment of Social Communication Profiles

Social communication deficits were examined using the La Trobe Communication Questionnaire (LCQ [30]). The LCQ is a 30-item questionnaire comprising statements relating to the frequency of social communication deficits in everyday life, for example, “When talking to others how frequently does s/he hesitate, pause or repeat themselves?” Carers rated the social communication profiles of patients, while controls completed a self-rated version of the questionnaire. A 4-point Likert scale was used to rate the frequency of behaviours (1, never; 2, sometimes; 3, often; 4, always), allowing for a total possible score of 120, with higher scores indicating greater perceived social communication deficits.

The LCQ further permits the assessment of social communication across 4 discrete subdomains: Initiation/Conversational flow, relating to initiating and maintaining conversation; Disinhibition/Impulsivity, pertaining to rude, embarrassing or inappropriate conversational attributes; Conversational Effectiveness, relating to logical, accurate and reciprocal conversation; and Partner Sensitivity, based on Gricean maxims of quality and quantity, i.e., communicative principles by which the quality and quantity of communication are as informative and effective as possible (see [47] for full details).

### 2.4. Statistical Analyses

Behavioural data were analysed using IBM SPSS Statistics (version 26). Prior to analyses, Shapiro–Wilks tests were used to check for normality of distributions across the variables of interest. Demographic and neuropsychological variables were investigated using one-way analysis of variance (ANOVA). Chi-squared tests were used to explore group differences in categorical variables (e.g., sex). A univariate analysis of variance (ANOVA) was used to investigate the main effects of groups (LPA, SD, PNFA, AD, bvFTD, controls) for total LCQ, while a multivariate ANOVA (MANOVA) was used to explore group effects across the LCQ subscales, followed by Sidak or Games–Howell post hoc tests as appropriate. Effect sizes for significant findings at *p* < 0.05 were reported using partial eta squared (η*_p_*^2^). One-tailed Pearson’s R correlations were used to explore associations between total LCQ performance and relevant clinical and neuropsychological variables.

### 2.5. Image Acquisition

Participants underwent whole-brain T_1_-weighted structural neuroimaging using a 3T Phillips MRI scanner with a standard quadrature head coil (eight channels). The 3D T_1_-weighted images were acquired via the following scanning sequences: coronal orientation, matrix 256 × 256, 200 slices, 1 mm^2^ in plane resolution, slice thickness = 1 mm, echo time/repetition = 2.6/5.8 ms and flip angle α = 8°. All scans were visually inspected for artefacts prior to analyses. Due to imaging contraindications (e.g., pacemaker, claustrophobia), structural scans were available for 7 LPA, 9 SD, 7 PNFA, 12 AD, 22 bvFTD and 22 controls (total imaging sample *n* = 79).

### 2.6. Voxel-Based Morphometry

Structural MRI data were analysed via voxel-based morphometry analyses (VBM) using the FSL-VBM toolbox from the FMRIB software package [48,49]. This technique permits the identification of voxel-by-voxel changes in grey matter intensity across the entire brain. Briefly, structural MR images were extracted using the brain extraction tool (BET) [50], following which tissue segmentation was conducted using FMRIB’s Automatic Segmentation Tool (FAST) [51]. The resultant grey matter partial volumes were then aligned to the Montreal Neurological Institute standard space (MNI52) via the FMRIB nonlinear registration tool (FNIRT) [52,53] using a b-spline representation of the registration warp field [54]. A study-specific template was created in which LPA, SD, PNFA, AD, bvFTD and control participants were equally represented to which the native grey matter images were re-registered nonlinearly. To correct for local expansion or contraction, the registered partial volume maps were modulated by dividing by the Jacobian of the warp field. Finally, the modulated and segmented images were smoothed using an isotropic Gaussian kernel with a sigma of 3 mm.

### 2.7. Profiles of Grey Matter Atrophy

Permutation-based non-parametric testing was used to investigate grey matter intensity differences between groups via an unbiased whole-brain general linear model [55] with 5000 permutations per contrast. Differences in cortical grey matter intensities between LPA, SD, PNFA, AD, bvFTD and Control participants were explored using regression models with separate directional contrasts (i.e., *t*-tests). Age was included as a nuisance variable in all contrasts. Clusters were extracted using the threshold free cluster enhancement method, corrected for family wise error (FWE) at *p* < 0.001. These analyses confirmed characteristic profiles of grey matter intensity decrease in the patient groups relative to controls (see Supplementary Material for full details).

### 2.8. Neural Substrates of Social Communication Changes

Finally, correlation analyses were run to explore associations between social communication dysfunction, as indexed by the total LCQ score, and grey matter intensity decrease across the entire brain. For this analysis, all participants were considered together as a single group (*n* = 79) to explore brain–behaviour relationships irrespective of clinical diagnosis. A general linear model with a negative t-contrast was run to explore associations between grey matter intensity decrease and higher total LCQ scores. Age was included as a nuisance variable in all contrasts. To boost our power to detect meaningful signal, while controlling for false positives, we extracted clusters voxel-wise and corrected using a false discovery rate of *q* = 0.05 [56]. This yielded a corrected *p*-value of 0.03 from the data. To further guard against false positives, statistical maps were thresholded using a strict cluster extent threshold of 50 contiguous voxels. Anatomical locations of statistical significance were overlaid on the MNI standard brain with maximum coordinates provided in the MNI stereotaxic space.

## 3. Results

### 3.1. Demographic and Neuropsychological Data

Table 1 displays background clinical and cognitive data for study participants. The participant groups did not differ in terms of age, sex, and education (all *p*-values > 0.16); and patient groups did not differ significantly for disease duration (years elapsed since symptom onset). Group differences were observed for disease severity (FRS) with AD and bvFTD patients exhibiting greater functional impairment compared with the PNFA group (*p*-values < 0.05). Significant group differences were evident in the ACE-III total scores (*F*(5,100) = 15.701, *p* < 0.0001, η*_p_*^2^ = 0.440), with LPA, SD, bvFTD and AD patient groups showing deficits in overall cognitive function relative to controls. No significant differences were observed in the ACE-III total between PNFA and Controls (*p* = 0.19). Games–Howell post hoc analyses revealed that bvFTD patients scored higher on the ACE-III in comparison to the LPA and AD groups (all *p*-values < 0.05), with no further between-patient group differences evident. Performance on the neuropsychological test battery revealed characteristic cognitive profiles in each dementia group in line with their clinical diagnoses (see Supplementary Material). Finally, carer-reported burden on the ZBI revealed an overall group effect (*F*(4,72) = 3.500; *p* = 0.011) driven by elevated carer burden in the bvFTD relative to PNFA carers, with no other group differences.

### 3.2. LCQ Performance

#### 3.2.1. Overall Social Communication Deficits

**Figure 1:**
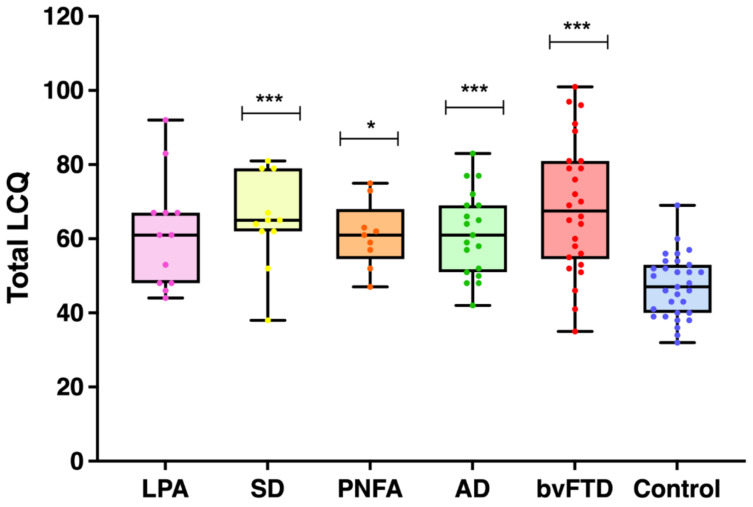
Distribution of total scores on the La Trobe Communication Questionnaire for all groups. Higher scores indicate greater social communication deficits. Asterisks denote significant group differences in relation to control scores. * *p* < 0.005, *** *p* < 0.001. Dots represent individual data points. Error bars represent minimum to maximum values.

Figure 1 and Table 2 display the results of the performance on the LCQ. A univariate ANOVA revealed a significant main effect of group on the LCQ (*F*(5,102) = 8.788, *p* < 0.0001; η*_p_*^2^ = 0.301). Games–Howell post hoc tests confirmed significantly higher LCQ total scores, indicating an overall reduction in social communication efficacy across the majority of patient groups relative to controls (bvFTD: *p* < 0.0001; AD: *p* = 0.001; SD: *p* = 0.007; PNFA: *p* = 0.013; LPA: *p* = 0.067) but no difference between the patient groups (all *p*-values > 0.5).

#### 3.2.2. Social Communication Profiles across Patient Groups

Looking across the LCQ subscales, a MANOVA revealed an overall effect of group on the following domains: Initiation/Conversational flow (*F*(5,102) = 8.824, *p* < 0.0001, η*_p_*^2^ = 0.302), Disinhibition/Impulsivity (*F*(5,102) = 4.058, *p* = 0.002, η*_p_*^2^ = 0.166), Conversational Effectiveness (*F*(5,102) = 3.484, *p* = 0.006, η*_p_*^2^ = 0.146) and Partner Sensitivity (*F*(5,102) = 5.431, *p* < 0.0001, η*_p_*^2^ = 0.210).

**Figure 2:**
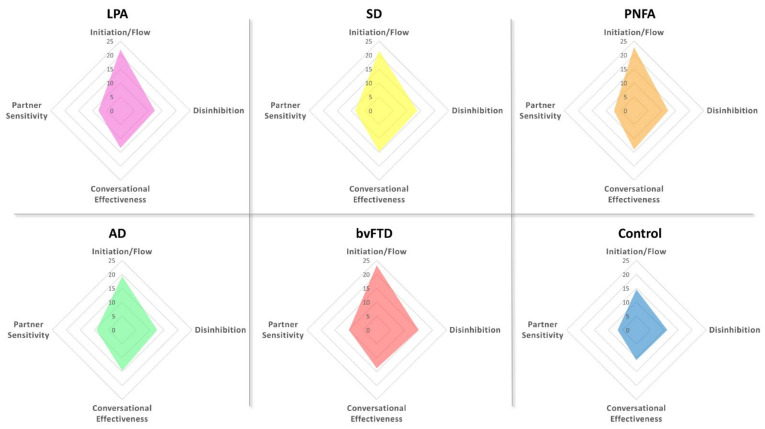
Radar charts showing social communication profiles on the La Trobe Communication Questionnaire subscales for each participant group. Higher scores indicate greater social communication deficits on that dimension.

Games–Howell post hoc tests revealed distinct social communication profiles in each patient group relative to controls (Table 2; Figure 2). Within the PPA cohort, the predominant social communication impairment reported by carers was that of reduced Initiation/Conversational flow on the LCQ. Relative to controls, LPA (*p* = 0.014) and PNFA (*p* = 0.002) were rated as displaying significantly impaired Initiation/Conversational flow in the context of relatively intact Disinhibition/Impulsivity, Conversational Effectiveness and Partner Sensitivity. A similar profile was evident for the SD group, with significantly compromised Initiation/Conversational flow (*p* = 0.028) as well as the suggestion of reduced Conversational Effectiveness (*p* = 0.058). AD patients were rated as significantly impaired in terms of Initiation/Conversational flow (*p* = 0.006), Conversational Effectiveness (*p* = 0.014) and Partner Sensitivity (*p* = 0.014), with no significant impairments in terms of Disinhibition/Impulsivity (*p* = 0.70). In contrast, bvFTD patients were rated as displaying impairments in Initiation/Conversational flow (*p* < 0.0001), Disinhibition/Impulsivity (*p* = 0.004) and Partner Sensitivity (*p* = 0.001), with relatively spared Conversational Effectiveness (*p* = 0.068).

#### 3.2.3. Correlations with Cognitive Function

One-tailed Pearson’s correlation analyses were run to explore associations between overall communication dysfunction and cognitive function in each patient group separately. Negative associations denote a significant relationship between cognitive decline and increased communication dysfunction on the LCQ total score. Considering the PPA syndromes first, LPA overall communication difficulties were negatively associated with global cognitive function (ACE-III total: *r* = −0.617 and *p* = 0.016), as well as single-word repetition (*r* = −0.549; *p* = 0.040), semantic association (*r* = −0.689; *p* = 0.010) and comprehension (*r* = −0.559; *p* = 0.037) on the SYDBAT. Similarly in SD, significant negative associations were evident between LCQ total and single-word repetition (*r* = −0.750; *p* = 0.010), semantic association (*r* = −0.557; *p* = 0.038) and comprehension (*r* = −0.568; *p* = 0.034). In contrast, PNFA total LCQ was significantly associated with overall cognitive dysfunction (ACE-III total: *r* = −0.757; *p* = 0.009). Collectively, these findings indicate that, unsurprisingly, social communication disruption is largely associated with overall language decline in PPA syndromes.

In AD, total LCQ was found to correlate exclusively with single-word repetition (SYDBAT: *r* = −0.525 and *p* = 0.013), while in bvFTD, significant associations were found between total LCQ and cognitive function (ACE-III total: *r* = −0.391; *p* = 0.024), single-word repetition (SYDBAT: *r* = −0.552; *p* = 0.009) and response inhibition (Hayling Total Cat A errors: *r* = 0.361; *p* = 0.045).

#### 3.2.4. Relationship between Social Communication Dysfunction and Carer Burden

One-tailed partial correlation analyses were run to explore the relationship between overall social communication dysfunction on the LCQ and patient functional outcomes on the FRS as well as carer-reported burden on the ZBI, controlling for overall disease duration. In LPA, social communication dysfunction was found to correlate with level of functional impairment on the FRS (*r* = −0.701; *p* = 0.008) but not with carer burden on the ZBI (*r* = 0.296; *p* = 0.189). The same profile of associations was observed in the PNFA group, whereby LCQ total was associated with functional impairment (FRS: *r* = −0.719; *p* = 0.022) but not with carer burden (ZBI: *r* = 0.507; *p* = 0.100). In contrast, LCQ correlated robustly with functional impairment (FRS: *r* = −0.806; *p* = 0.002) and carer burden (ZBI: *r* = 0.811; *p* = 0.002) in the SD group. The same pattern of associations was evident in bvFTD (FRS: *r* = −0.677; *p* = 0.001 and ZBI: *r* = 0.721; *p* < 0.0001), while no significant associations were found in AD (all *p*-values > 0.070).

### 3.3. Neuroimaging Analyses

**Figure 3:**
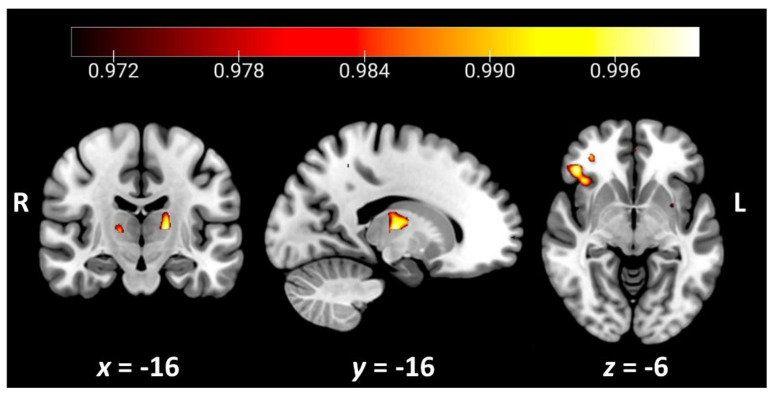
Grey matter correlates of overall social communication dysfunction on the LCQ total across the entire study cohort (*n* = 79). Coloured voxels indicate regions that emerged as significant in the voxel-based morphometry analyses, extracted voxel-wise and corrected for a false discovery rate of *q* = 0.05 (corrected *p* < 0.03). All clusters reported *t* > 2.67. Age was included as a nuisance variable in the analyses. Clusters are overlaid on the Montreal Neurological Institute (MNI) standard brain with *x*- and *y*-coordinates reported in the MNI standard space. L = left; R = right. Figures created using MRIcroGL.

Regions of decreased grey matter intensity associated with higher total scores on the LCQ are presented in Table 3 and Figure 3. The overall severity of social communication dysfunction was associated with grey matter intensity decrease in a discrete set of regions, including the right orbitofrontal cortex extending into the insular cortex, the right inferior frontal gyrus, the right frontal pole, and the left thalamus.

## 4. Discussion

The objective of this study was to explore multidimensional profiles of social communication in PPA using the La Trobe Communication Questionnaire (LCQ) and to identify potential cognitive and neural mechanisms driving these changes. Overall, distinct changes in social communication were observed in PNFA and SD, with a trend towards impairment in the LPA group. These deficits reflect a decline in pragmatics and the use of language, both verbal and non-verbal, in social contexts. Deficits in social communication of the same magnitude as that displayed in PPA were also observed in the AD and bvFTD groups, suggesting that a breakdown in socioemotional aspects of communication may be a transdiagnostic feature of neurodegenerative disorders. Looking at specific social communication profiles, initiation and conversational flow were found to be uniformly impaired irrespective of dementia subtype and may reflect a domain-general marker of dementia. Despite this shared feature, disease-specific impairments emerged across the LCQ subscales, suggesting that the canonical language and speech production disturbances displayed by PPA patients on formal clinical assessment manifest in variable ways in their everyday discourse.

Considering first the PPA syndromes, PNFA and LPA were reported to display significant impairments exclusively in initiation and conversational flow. This finding is largely in keeping with the canonical motor speech disturbances in PNFA and word-finding difficulty and conversational lapses characteristic of LPA [2]. While overall communication difficulties were associated with degree of cognitive impairment on the ACE-III in both groups, LPA social communication difficulties were also associated with indices of language dysfunction, including single-word repetition, semantic association and semantic comprehension. These differential correlations speak to the multifaceted nature of social communication changes in PPA and the fact that behaviourally similar profiles can emerge due to the breakdown of distinct underlying cognitive, linguistic and motoric processes.

Interestingly, we did not find significant associations between overall social communication dysfunction and perceived burden in carers of LPA or PNFA patients; however, robust correlations were found with functional impairment in both syndromes. Accordingly, while social communication difficulties impinge negatively on everyday functional activities in these patients, they do not appear to be viewed as burdensome by carers and may reflect their relatively preserved capacity to connect socially in certain domains. The experience of PPA has been shown to vary widely depending on subtype and patient/carer perspective, whereby individuals with PPA tend to focus on language decline while family members concentrate their efforts on adapting to and managing socioemotional and behavioural changes [57]. When viewed in this light, the types of communication dysfunction assessed by the LCQ are likely to be anticipated by carers as part of the typical disease trajectory in PNFA and LPA and perceived as more manageable. What remains unclear is how these changes impact the patient’s lived experience and sense of self [58], and we suggest that this is a critical area for future empirical research.

Turning our attention to the communication profile of SD, we found evidence of disrupted initiation and conversational flow, with a trend towards reduced conversational effectiveness in this syndrome (see also [59]). Intuitively, these features make sense within the overall cognitive landscape of SD, which is dominated by the deterioration of core semantic processes that are central to the production of content-rich speech [60]. Previous studies suggest prominent changes in the content of narrative discourse in SD, with patients defaulting to the present tense and less complex narrative structure during autobiographical narration [61]. Moreover, with worsening semantic impairment, SD patients have been observed to become increasingly rigid in terms of their preferences, behaviours, and creative problem-solving capacity [62,63,64]. It remains unclear how the progressive narrowing of the conceptual knowledge space impacts topic selection and maintenance during natural discourse. However, our correlation analyses indicated significant associations between overall social communication dysfunction in SD and independent clinical assessments of semantic processing. In contrast to PNFA and LPA, the impact of social communication disturbances in SD was evident both in terms of patient functional impairment and level of carer burden. This finding resonates strongly with a recent qualitative review of the lived experience in PPA in which carers of SD reported higher levels of perceived burden, which were largely driven by behavioural changes and rigidity in this syndrome [57]. Reduced conversational effectiveness in SD due to hallmark features of anomia and circumlocutions may be perceived by carers as topic derailment or oppositional behaviour (see also [19]). This proposal dovetails with findings of off-target verbosity during autobiographical narration in SD, where patients default to personally relevant and familiar topics drawn from their intact episodic memory store [65,66,67], inadvertently compromising the effectiveness of their interactions. Emotion recognition may also play an important modulating role in this context, as studies suggest that verbosity in older age may, in part, reflect an inability to decode emotional cues of the listener [68]. Changes in emotion processing and empathy are increasingly recognised in SD and have been shown to impact negatively on the quality of the patient–carer relationship [11,19,20,69]. It remains unclear how emotion processing difficulties relate to social communication changes in SD. However, given the negative impact of social communication dysfunction on patient and carer outcomes, this represents an important area for future study to inform targeted interventions.

Disease-specific changes in social communication were also evident in the non-PPA syndromes. Briefly, in addition to impaired initiation and conversational flow, AD patients displayed reduced conversational effectiveness and partner sensitivity. On the surface, reduced partner sensitivity would appear somewhat at odds with reports of relatively intact socioemotional processing in AD, at least in the early stages of the disease course [19,70]. Closer inspection of the partner sensitivity index, however, reveals items that likely tax dynamic aspects of conversational discourse related to episodic and working memory functions, manifesting in repetitive content and topic switching that could be perceived as insensitive to the conversational partner. Importantly, despite carers rating an increase in social communication difficulties in AD, this was not associated with functional impairment in patients or with perceived burden in carers. In contrast, patients with bvFTD were reported to display disinhibition and diminished partner sensitivity alongside impaired initiation and conversational flow. These disturbances are in keeping with the profound socioemotional impairments in empathy, theory of mind, emotion processing and stimulus-bound thinking that typify the bvFTD syndrome [22,71,72,73]. While bvFTD patients do not display an obvious aphasia, they do display difficulties with prosody, comprehension and expression of abstract words and narratives [74]. Collectively, these cognitive and social comportment changes impact dramatically on the capacity to engage effectively in conversational discourse and may compound aspects of functional decline and carer burden in this syndrome.

A secondary aim of this study was to explore the potential underlying neural substrates of social communication changes in dementia using a transdiagnostic approach. Our voxel-based morphometry analyses revealed significant associations between increased social communication disturbances and grey matter intensity decrease in discrete cortical and subcortical regions, including right-sided orbitofrontal and insular cortices, inferior frontal gyrus, and the left thalamus. The finding of significant right-sided orbitofrontal and frontoinsular cortical involvement resonates with an expansive literature implicating these structures in complex cognitive, affective and interoceptive processes that are essential for higher-level social cognitive functioning and behavioural regulation [75,76], as well as for assigning value to stimuli and in reward processing broadly [77,78]. These regions exhibit strong connections to the inferior frontal gyrus, which supports attentional control, response inhibition and aspects of speech and social cognitive function [79] and emerged as a significant neural correlate in our analyses. Finally, we found evidence of significant thalamic involvement, resonating with an emerging literature in which the thalamus is viewed as making an important contribution to cognition [80]. Mounting reports indicate that rather than being a passive relay station, the thalamus is actively involved in integrative cognitive processes, including learning and memory, flexible adaptation, and the shaping of mental representations [81]. Our findings, therefore, suggest that disruption of a right-sided cortico-thalamic circuit may contribute to social communication disturbances seen across dementia syndromes.

A number of methodological considerations and future directions warrant discussion. First, our sample size is relatively small, reflecting the rarity of the PPA syndromes of interest. Our findings warrant replication in a larger cohort ideally stratified by disease staging and symptom severity to understand the prevalence and timing of these social communication difficulties. Second, while informant-based questionnaires are a useful tool to examine the inherently collaborative nature of social communication, it is imperative to understand the lived experience of the patient in this regard. A limitation of our study is that we compared carer reports for patients with self-reports for controls. Ideally, future studies should collect self-ratings and informant ratings of social communication for all participants to ensure the comparability of findings. A key issue will be to determine whether these changes in social communication represent a source of stress for the patient and the extent to which these changes are disruptive in terms of the functional goals of communication. Future studies could complement the data provided by the LCQ by incorporating naturalistic discourse assessments to determine the experience of patients when engaging in communication across different social settings. In this regard, conversation analysis may prove particularly fruitful in providing direct insights into the social communication profiles of people with PPA, as well as the evolution of these profiles over time. Given mounting evidence of distinct cognitive and behavioural trajectories with advancing disease severity in PPA [7,82], longitudinal studies will provide crucial information to inform the nature of therapeutic support required by patients over time. In addition, it will be important for future studies to explore how impairments in social cognitive processes, such as theory of mind, empathy, and mental simulation, are potentially impacted by, and interact with, canonical language disturbances in dementia syndromes. Similarly, an interesting further consideration is whether motivational disturbances mediate changes in social communication, given emerging evidence of apathy (i.e., decreased goal-directed behaviour) and anhedonia (i.e., decreased interest in and response to rewarding experiences) in PPA [83,84,85,86]. Whether patients with dementia become less motivated to engage in activities involving social communication represents an unanswered question that will be crucial to address prior to trialling interventions.

## 5. Conclusions

In summary, we have provided evidence of social communication disruption in PPA and non-PPA syndromes, with variable profiles of social communication observed across the patient groups. Our findings hold a number of important clinical implications. While PPA syndromes predominantly displayed impaired initiation and conversational flow, partner sensitivity and disinhibition did not seem to be compromised. Understanding such profiles of loss and sparing in conversational discourse is important to identifying potential strategies to support patient–carer communication and alleviate stress or burden associated with these changes [87]. For example, communication training programs have been successfully implemented to improve turn-taking and decrease verbosity during conversational discourse in patients with traumatic brain injury (TBI, e.g., [88]). Similarly, communication partner training programmes have been shown to provide an effective means of targeting distinct aspects of communication in various neurological disorders, including stroke, TBI, and dementia [89,90]. Translating such approaches for use in the PPA setting will, therefore, be an important future direction for this research.

## Figures and Tables

**Table 1:** Demographic and clinical characteristics of the study sample.

Demographics	LPA (*n* = 12)	SD (*n* = 11)	PNFA (*n* = 9)	AD (*n* = 19)	bvFTD (*n* = 26)	Control (*n* = 31)	*F*-Test	Post Hoc
Age (y)	69.8 (7.0)	63.8 (6.2)	67.1 (6.1)	66.1 (7.6)	64.8 (5.8)	65.4 (6.3)	NS	-
Education (y)	11.9 (3.5)	13.8 (2.3)	12.4 (3.2)	13.2 (3.7)	12.1 (3.7)	13.9 (3.7)	NS	-
Sex (M:F)	7:5	7:4	2:7	10:9	19:7	16:15	NS	-
Disease Duration (y)	5.2 (2.5)	6.7 (2.9)	6.1 (3.0)	7.2 (5.1)	7.5 (4.7)	-	NS	-
ACE-III Total (100)	63.7 (17.1)	71.1 (21.7)	80.0 (16.1)	69.6 (12.5)	82.8 (9.3)	94.1 (3.8)	***	CN > Patients
FRS (Rasch score)	0.85 (1.4)	0.82 (1.4)	2.7 (1.8)	0.42 (1.1)	−0.38 (1.3)	-	***	PNFA > AD, bvFTD
ZBI (48)	13.2 (6.8)	18.7 (8.7)	11.4 (6.5)	15.2 (8.7)	21.4 (10.0)	-	*	bvFTD > PNFA

Notes: Means with standard deviations in parentheses. AD = Alzheimer’s disease; bvFTD = behavioural variant of frontotemporal dementia; CN = controls; LPA = logopenic progressive aphasia; PNFA = progressive non-fluent aphasia; (y) = years; ZBI = Zarit Burden Interview. * *p* < 0.05; *** *p* < 0.0001; - = not applicable; NS = not significant. Education data available for 28 controls. Disease duration available for 15 AD. ACE-III data available for 29 controls. FRS data available for 14 AD and 20 bvFTD.

**Table 2:** Breakdown of social communication difficulties on the La Trobe Communication Questionnaire.

LCQ Score	LPA (*n* = 12)	SD (*n* = 11)	PNFA (*n* = 9)	AD (*n* = 19)	bvFTD (*n* = 26)	Control (*n* = 31)	*F*-Test	Post Hoc
Total (120)	61.4 (15.0)	64.9 (12.5)	61.0 (9.0)	61.5 (11.4)	68.6 (17.8)	47.1 (8.4)	***	CN < LPA, SD, AD, bvFTD
Initiation/Flow	22.0 (6.1)	21.6 (6.0)	22.8 (4.2)	19.3 (4.6)	23.2 (7.6)	14.5 (3.2)	***	CN < Patients
Disinhibition	12.3 (3.3)	13.7 (3.0)	12.2 (2.4)	12.5 (3.8)	15.0 (4.6)	11.1 (2.3)	**	CN < bvFTD
Conversational Effectiveness	13.4 (4.3)	14.7 (4.3)	13.9 (2.2)	14.6 (3.2)	13.7 (4.2)	10.8 (3.6)	**	CN < AD
Partner Sensitivity	7.9 (2.9)	8.6 (2.5)	7.2 (1.7)	9.2 (2.7)	9.9 (3.2)	6.7 (1.6)	***	CN < AD, bvFTD

Notes: Scores presented as means, with standard deviations in parentheses. The maximum test score provided in brackets, where higher scores denote poorer social communication function. AD = Alzheimer’s disease; bvFTD = behavioural variant of frontotemporal dementia; CN = controls; LCQ = La Trobe Communication Questionnaire; LPA = logopenic progressive aphasia; PNFA = progressive non-fluent aphasia. *** *p* < 0.0001; ** *p* < 0.01.

**Table 3:** Grey matter correlates of perceived social communication dysfunction across the entire study cohort (*n* = 79).

Contrast	Regions	Side	Cluster Size	Cluster Peak MNI Coordinates			*t*-Value
				x	y	z	
LCQ Total	Orbitofrontal cortex, insular cortex, inferior frontal gyrus	R	204	44	32	−6	3.20
	Thalamus	L	113	−16	−16	6	3.09
	Frontal pole	R	57	32	64	2	2.67

Notes: Age was included as a nuisance variable in all contrasts. Clusters are reported voxel-wise, corrected for a false discovery rate of *q* = 0.05 (corrected *p* = 0.03) and a cluster extent threshold of 50 contiguous voxels. MNI = Montreal Neurological Institute; L = left; R = right.

## Data Availability

The ethical requirement to ensure patient confidentiality precludes public archiving of our data. Researchers who would like to access the raw data should contact the corresponding author, who will liaise with the ethics committee that approved the study. Accordingly, as much data as are required to reproduce the results will be released to the individual researcher. No parts of the study procedures or analyses were registered prior to the research being undertaken.

## Supplementary Materials

The following are available online at https://www.mdpi.com/article/10.3390/brainsci11121600/s1: Table S1: Cognitive performance across neuropsychological domains of interest; Table S2: Profiles of grey matter intensity decrease in patient groups relative to controls.
