# Supplementary material for: Cognitive and Neural Mechanisms of Social Communication Dysfunction in Primary Progressive Aphasia
Source: Brain Sci. 2021 Dec 1;11(12):1600. doi: 10.3390/brainsci11121600 (PMC8699060; doi:10.3390/brainsci11121600)
Supplement: Supplementary file 1 [file brainsci-11-01600-s001.zip › brainsci-1467662-supplementary.pdf]

Supplementary Material.

Cognitive and neural mechanisms of social communication dysfunction in Primary Progressive Aphasia

Zoë-Lee Goldberg [1,2], Hashim El-Omar [1,2], David Foxe [1,2,3], Cristian E. Leyton [1,2], Rebekah M. Ahmed [1,3,4], Olivier Piguet [1,2], and Muireann Irish [1,2*]

1 The University of Sydney, Brain and Mind Centre, Sydney, New South Wales, 2050, Australia; zoe.goldberg@sydney.edu.au (Z.L.G.)
2 The University of Sydney, School of Psychology, Sydney, New South Wales, 2006, Australia; hashim.el-omar@sydney.edu.au (H.E.O.), david.foxe@sydney.edu.au (D.F.), cristian.leyton@sydney.edu.au (C.L.), olivier.piguet@sydney.edu.au (O.P.)
3 University of Sydney, School of Medical Sciences, Sydney, New South Wales, 2006, Australia; rebekah.ahmed@sydney.edu.au (R.M.A.)
4 Memory and Cognition Clinic, Department of Clinical Neurosciences, Royal Prince Alfred Hospital, Sydney, NSW, 2050, Australia
* Correspondence: muireann.irish@sydney.edu.au; Tel.: +61 2 9114 4165

Files included:

Table S1. Cognitive performance across neuropsychological domains of interest.
Table S2. Profiles of grey matter intensity decrease in patient groups relative to controls.

Table S1. Cognitive performance across neuropsychological domains of interest

Cognitive domain	LPA (*n* = 12)	SD (*n* = 11)	PNFA (*n* = 9)	AD (*n* = 19)	bvFTD (*n* = 26)	Controls (*n* = 31)	F test	Post hoc
ACE-III Attention [18]	11.5 (4.2)	15.7 (3.0)	15.8 (2.4)	12.6 (3.6)	16.0 (2.3)	17.3 (.90)	***	CN > LPA, AD
ACE-III Memory [26]	14.6 (6.3)	16.7 (7.3)	21.9 (6.4)	13.4 (4.2)	20.4 (4.2)	24.5 (1.8)	***	CN > bvFTD, SD, AD, LPA
ACE-III Fluency [14]	4.8 (3.1)	7.6 (3.6)	6.7 (3.9)	7.1 (3.6)	8.0 (2.7)	11.9 (1.7)	***	CN > Patients
ACE-III Language [26]	19.4 (4.6)	15.91 (7.7)	21.33 (3.3)	23.1 (3.0)	23.7 (2.2)	25.0 (1.3)	***	CN > LPA, SD
ACE-III Visuospatial [16]	13.5 (1.9)	15.1 (1.6)	14.3 (2.2)	13.5 (2.1)	14.9 (1.5)	15.5 (.95)	***	CN > LPA, AD
Digit Span Forward	6.9 (2.3)	10.2 (2.5)	8.2 (2.8)	8.6 (2.0)	10.3 (2.8)	11.2 (2.2)	***	CN > PNFA, AD, LPA
Trail Making Test Part B-A [secs]	146.0 (127.7)	55.2 (50.9)	105.3 (50.3)	161.9 (128.5)	72.0 (66.4)	41.3 (16.7)	***	CN < LPA, AD
RCF Copy [36]	26.1 (9.6)	30.5 (3.0)	26.8 (3.7)	24.1 (7.7)	28.7 (6.5)	31.2 (4.1)	**	CN > AD
RCF Percent Retained [%]	38.3 (22.7)	46.4 (24.4)	47.5 (16.0)	15.7 (15.4)	41.3 (23.5)	51.6 (11.4)	***	CN, PNFA, SD, bvFTD > AD
SYDBAT Naming [30]	17.2 (6.9)	12.6 (8.5)	20.8 (8.0)	21.6 (4.4)	23.3 (3.9)	27.0 (2.3)	***	CN > PNFA, AD, LPA, SD
SYDBAT Comprehension [30]	26.4 (2.5)	21.2 (8.4)	28.6 (1.3)	26.2 (2.7)	27.8 (2.0)	29.1 (1.2)	***	CN > AD, SD
SYDBAT Semantic Association [30]	24.2 (5.5)	19.7 (8.9)	25.4 (4.1)	25.4 (2.9)	27.1 (2.1)	28.4 (1.1)	***	CN > SD

SYDBAT Repetition [30]	25.6 (6.1)	29.4 (.9)	17.4 (10.7)	29.3 (.91)	29.1 (1.2)	29.8 (.4)	***	CN > LPA, PNFA
Hayling Category A Errors	1.5 (3.0)	2.5 (.7)	1.5 (.6)	2.7 (1.9)	2.7 (3.7)	.79 (.8)	NS	
Hayling Category B Errors	2.0 (.8)	2.0 (2.8)	1.8 (1.5)	3.5 (1.6)	3.5 (2.3)	1.8 (2.3)	NS	

Notes: Maximum test scores provided in square brackets where appropriate. AD = Alzheimer's disease; bvFTD = behavioural variant of frontotemporal dementia; CN = Controls; LPA = logopenic progressive aphasia; PNFA = progressive non-fluent aphasia; RCF = Rey Complex Figure; SD = semantic dementia. ** $p < .010$; ***$p < .001$; - = not applicable; NS = not significant.
ACE-III data available for 29 Controls; Digit Span data available for 28 Controls; Trail Making Test data available for 14 AD, 22 bvFTD, 28 Controls, 7 LPA, 10 SD and 7 PNFA; RCF Copy data available for 16 AD, 25 bvFTD, 26 Controls, and 11 LPA; RCF Percent Retained data available for 16 AD, 25 bvFTD, 26 Controls, 11 SD and 10 LPA; SYDBAT Naming data available for 18 bvFTD, 28 Controls, 11 LPA and 8 PNFA; SYDBAT Comprehension data available for 18 bvFTD, 28 Controls, 11 LPA and 8 PNFA; SYDBAT Semantic Association data available for 18 AD, 18 bvFTD, 28 Controls, 11 LPA, 11 SD and 8 PNFA; SYDBAT Repetition data available for 18 AD, 18 bvFTD, 28 Controls, 11 LPA, 9 SD and 8 PNFA; Hayling data available for 15 AD, 23 bvFTD, 28 Controls, 4 LPA, 2 SD and 4 PNFA.

Table S2. Profiles of grey matter intensity decrease in patient groups relative to controls.

Contrast relative to controls	Regions	Side	Cluster size	Peak MNI coordinates *x y z*
LPA	Frontal orbital cortex, temporal pole, superior temporal gyrus, insular cortex, parietal operculum cortex, Heschl's gyrus, central opercular cortex, parietal operculum cortex, supramarginal gyrus	L	3,783	-20 12 -16
SD	Temporal pole, temporal fusiform cortex, insular cortex, amygdala, putamen parahippocampal gyrus, hippocampus, orbitofrontal cortex	R	2,241	34 6 -40
	Temporal pole, insular cortex, planum polare, amygdala, hippocampus, putamen	L	1,346	-36 10 -30
PNFA	*No significant clusters*			
AD	Temporal pole, inferior/superior temporal gyrus, parahippocampal gyrus, hippocampus, putamen, caudate, amygdala, orbitofrontal cortex, frontal pole, insular cortex, precentral gyrus, postcentral gyrus, inferior/middle frontal gyrus, Heschl's gyrus, supramarginal gyrus, angular gyrus.	R	9,824	44 20 -40
	Temporal pole, orbitofrontal cortex, insular cortex, putamen, pallidum, amygdala, hippocampus, parahippocampal gyrus.	L	3,497	-32 2 -30
	Planum temporale, middle/superior temporal gyrus, parietal operculum cortex, angular gyrus, supramarginal gyrus, lateral occipital cortex.	L	2,361	-54 -24 4
	Left precuneus, left lateral occipital cortex, right precuneus, right postcentral gyrus, right lateral occipital cortex	B	1,348	-6 - 58 50
	Posterior cingulate gyrus, precuneus, lateral occipital cortex	L	535	-16 -36 40
	Lateral occipital cortex, middle temporal gyrus, angular gyrus	R	509	48 -60 8
	Posterior cingulate gyrus, hippocampus, thalamus, parahippocampal gyrus	R	378	22 -42 0
	Supramarginal gyrus, angular gyrus	L	268	-54 -50 42

bvFTD	Right inferior temporal gyrus, right temporal pole, right frontal pole, right orbitofrontal cortex, right insular cortex, right hippocampus, right amygdala, right putamen, right caudate, right pallidum, right parahippocampal gyrus, right thalamus, right Heschl's gyrus, right supramarginal gyrus, right angular gyrus, right lateral occipital cortex, right superior temporal gyrus, superior frontal gyrus, right paracingulate gyrus, left paracingulate gyrus, left superior frontal gyrus, left anterior cingulate gyrus, left frontal pole, left orbitofrontal cortex, left temporal pole, left insular cortex, left putamen, left caudate, left amygdala, left hippocampus, left parahippocampal gyrus, left cerebellum, left thalamus, left supramarginal gyrus, left angular gyrus, left lateral occipital cortex	B	33,185	46 2 -46

Note. Age included as a nuisance variable in all contrasts. Clusters were extracted using the threshold-free cluster enhancement method and corrected for Family-Wise Error at $p < .001$. All clusters reported $t > 3.71$. MNI = Montreal Neurological Institute; L = Left; R = Right; B = Bilateral.
